# Cardiomyopathies and myocardial disorders in Africa: present status and the way forward

**Published:** 2013-04

**Authors:** Bongani M Mayosi

**Affiliations:** Department of Medicine, Old Groote Schuur Hospital, Cape Town, South Africa

## Dear Sir

I read with great interest the review by Falase and Ogah on cardiomyopathies and myocardial disorders in Africa.^1^ It is a timely contribution to the ongoing discourse on the contemporary status of heart muscle disease in Africa.^2^,^3^ There are however several issues that need to be addressed by the authors of the review. The first relates to the statement by the authors that ‘there are no reports of left ventricular non-compaction from Africa, possibly because African cardiologists are not yet familiar with its echocardiographic changes’. This statement is contrary to the published literature. Over the past six years, there have been several reports from different countries of African patients with left ventricular non-compaction, including Djibouti, South Africa and Sudan.^4^-^8^

The second issue relates to the following statement in the abstract and text of the review: ‘there are no reports of … ion channelopathies in Africa’. I would like to draw the authors to the discovery of impaired endocytosis of the ion channel TRPM4 as a cause of human progressive familial heart block type I in South Africans.^9^ This work by colleagues and their collaborators from Stellenbosch University represents one of the most significant contributions of African scientists to the understanding of the pathogenesis of cardiac disease in recent times.

The third issue is one of clarification. The authors refer to genotyping the ‘Hb’ gene in patients with cardiomyopathy. It is not clear what the ‘Hb’ gene is, or the rationale for postulating a linkage with cardiomyopathy. Information on the locus on the gene map and laboratory conditions used for typing the gene would assist other investigators in verifying the findings of the authors.

The fourth issue from the review relates to the discussion of the classification of cardiomyopathies. The authors propose a new classification that is based on the proposal of the American Heart Association.^10^ It is curious that the authors have omitted any reference to the alternative classification scheme of the European Society of Cardiology.^11^ In the latter scheme, cardiomyopathy is regarded as a structural and functional abnormality of the myocardium that is not due to hypertension, coronary artery disease, valvular heart disease, pericardial disease, or congenital heart disease. Furthermore, cardiomyopathy is sub-classified into familial/genetic or non-familial/non-genetic types.

I have found that the European Society of Cardiology classification lends itself well to the clinical evaluation of patients with unexplained heart failure in the African setting.^12^,^13^ It would be of interest to know the opinion of the authors and that of the Pan-African Society of Cardiology (as suggested by the authors) on the utility of the European classification of cardiomyopathy compared to the version of the American Heart Association in the African environment.

Finally, the authors make a case for a new and unique classification of myocardial disorders for Africa. It is not clear why Africans should be an exception to other populations of the world. We have shown previously that while the burden of disease may be higher for certain forms of cardiomyopathy in Africa, the pathophysiological features of the cardiomyopathies are likely to be the same in all continental populations.^13^,^14^ Therefore, the aspiration of the Pan-African Society of Cardiology should probably be to contribute to the development of a universal classification of cardiomyopathy for all people in the world, possibly under the auspices of the World Health Organisation or the World Heart Federation.

## References

[1] Falase AO, Ogah OS (2012). Cardiomyopathies and myocardial disorders in Africa: present status and the way forward.. Cardiovasc J Afr.

[2] Sliwa K, Damasceno A, Mayosi BM (2005). Epidemiology and etiology of cardiomyopathy in Africa.. Circulation.

[3] Mayosi BM (2007). Contemporary trends in the epidemiology and management of cardiomyopathy and pericarditis in sub-Saharan Africa.. Heart.

[4] Ker J, Van Der Merwe C (2006). Isolated left ventricular non-compaction as a cause of thrombo-embolic stroke: a case report and review.. Cardiovasc J S Afr.

[5] Ali SK (2008). Unique features of non-compaction of the ventricular myocardium in Arab and African patients.. Cardiovasc J Afr.

[6] Massoure PL, Lamblin G, Bertani A, Eve O, Kaiser E (2011). Rare cause of heart failure in an elderly woman in Djibouti: left ventricular non compaction.. Med Trop (Mars).

[7] Peters F, Dos Santos C, Essop R (2011). Isolated left ventricular non-compaction with normal ejection fraction.. Cardiovasc J Afr.

[8] Peters F, Khandheria BK, dos Santos C, Matioda H, Mogogane MT, Essop MR (2012). Isolated left ventricular noncompaction in identical twins.. Am J Cardiol.

[9] Kruse M, Schulze-Bahr E, Corfield V (2009). Impaired endocytosis of the ion channel TRPM4 is associated with human progressive familial heart block type I.. J Clin Invest.

[10] Maron BJ, Towbin JA, Thiene G (2006). Contemporary definitions and classification of the cardiomyopathies: An American Heart Association scientific statement from the Council on Clinical Cardiology, Heart Failure and Transplantation Committee; Quality of Care and Outcomes Research and Functional Genomics and Translational Biology Interdisciplinary working groups; and Council on Epidemiology and Prevention.. Circulation.

[11] Elliott P, Andersson B, Arbustini E (2008). Classification of the cardiomyopathies: a position statement from the European Society of Cardiology working group on myocardial and pericardial diseases.. Eur Heart J.

[12] Ntusi NBA, Badri M, Gumedze F, Wonkam A, Mayosi BM (2011). Clinical characteristics and outcomes of familial and idiopathic dilated cardiomyopathy in Cape Town: A comparative study of 120 cases followed up over 14 years.. S Afr Med J.

[13] Ntusi NBA, Wonkam A, Shaboodien G, Badri M, Mayosi BM (2011). Frequency and clinical genetics of familial dilated cardiomyopathy in Cape Town: Implications for the evaluation of patients with unexplained cardiomyopathy.. S Afr Med J.

[14] Watkins DA, Hendricks N, Shaboodien G (2009). Clinical features, survival experience, and profile of plakophylin-2 gene mutations in participants of the Arrhythmogenic Right Ventricular Cardiomyopathy Registry of South Africa.. Heart Rhythm.

